# Durable response to nivolumab in combination with regional hyperthermia in a patient with PD-L1-negative metastatic head and neck squamous cell carcinoma

**DOI:** 10.1007/s00262-025-04029-9

**Published:** 2025-04-17

**Authors:** Luc M. Berclaz, Anton Burkhard-Meier, Axel Lechner, Michael Völkl, Sinan E. Güler, Sultan Abdel-Rahman, Sina Mansoorian, Wolfgang G. Kunz, Thomas Knösel, Martin Canis, Michael von Bergwelt-Baildon, Rolf D. Issels, Dorit Di Gioia, Lars H. Lindner

**Affiliations:** 1 Department of Internal Medicine III, University Hospital, LMU Munich, Munich, Germany; 2 German Cancer Consortium (DKTK), Partner Site Munich, Munich, Germany; 3 Deparment of Otorhinolaryngology, University Hospital, LMU Munich, Munich, Germany; 4 Department of Radiation Oncology, University Hospital, LMU Munich, Munich, Germany; 5 Department of Radiology, University Hospital, LMU Munich, Munich, Germany; 6 Institute of Pathology, LMU Munich, Munich, Germany

**Keywords:** Immunotherapy, Immune checkpoint inhibitors, Head and neck cancer, Regional hyperthermia

## Abstract

We report a long-lasting response to the immune checkpoint inhibitor nivolumab in combination with regional hyperthermia (RHT) in a patient with recurrent metastatic Head and Neck Squamous Cell Carcinoma (HNSCC) and negative programmed death ligand 1 (PD-L1) expression. Treatment was well tolerated with no local side effects. Tumor-related symptoms in the orbital and masticator area gradually decreased under treatment with nivolumab and RHT. Over the course of treatment, magnetic resonance imaging (MRI) showed a local tumor control in the heated tumor areas, while metastatic lesions developed in areas outside of the RHT field. This is the first case report demonstrating the feasibility and clinical potential of the addition of RHT in this patient collective with poor outcomes and low response rates to immune checkpoint inhibitors. RHT might be an additional tool to activate an immunogenic milieu responsive to immune checkpoint inhibitors.

## Background

Prognosis is exceptionally poor in metastatic Head and Neck Squamous Cell Carcinoma (HNSCC), and therapeutic options are limited in patients with recurrent disease. Immune checkpoint inhibitors target checkpoint mediators such as programmed death ligand 1 (PD-L1) and have shown to be effective in several randomized trials on HNSCC [1–3]. They are now part of international guidelines as an additional therapy line in combination or after failure of conventional chemotherapy. Response rates in patients with negative PD-L1 expression are very limited [4–6]. Regional hyperthermia (RHT) has shown to be a potent immune inductor and has the potential to increase the efficacy of systemic therapy [7, 8]. While RHT is an established treatment modality in combination with chemo- and radiotherapy in several solid tumors [7, 9], data on the combination of RHT with immune checkpoint inhibitors are currently limited to preclinical data [10]. This is the first case report to demonstrate a prolonged response to nivolumab in combination with RHT in a patient with metastatic HNSCC and negative PD-L1 expression.

## Case presentation

We present the case of a patient who was first diagnosed with oropharyngeal squamous cell carcinoma in 2018. Magnetic resonance imaging (MRI) staging demonstrated a tumor manifestation in the base of the tongue on the left side, extending to the lingual surface of the epiglottis. Initial stage was cT4 cN2 cM0. Histological work-up demonstrated a p16-positive squamous cell carcinoma. Staining for PD-L1 was negative (TPS 0%, CPS 0, IC-Score 0%) (Ventana SP263 PD-L1 assay [11]). Due to the locally advanced stage, the patient received definitive radiochemotherapy. A dose of 1.8 Gray (Gy) was delivered by volumetric-modulated arc therapy (VMAT) in fractions, for a total of 50.4 Gy to the tumor and cervical lymph node stations, with a boost to 59.4 Gy for the pathological lymph nodes and up to 70.2 Gy for the tumor region. Simultaneously, the patient received cisplatin (40 mg/m^2^ BSA) for two cycles. Chemotherapy with cisplatin was permanently discontinued after two cycles due to acute kidney injury.

**Fig. 1:**
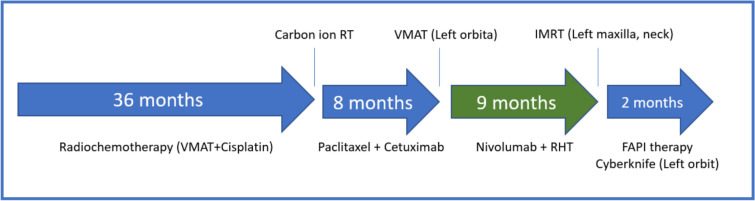
Successive treatment lines

**Fig. 2:**
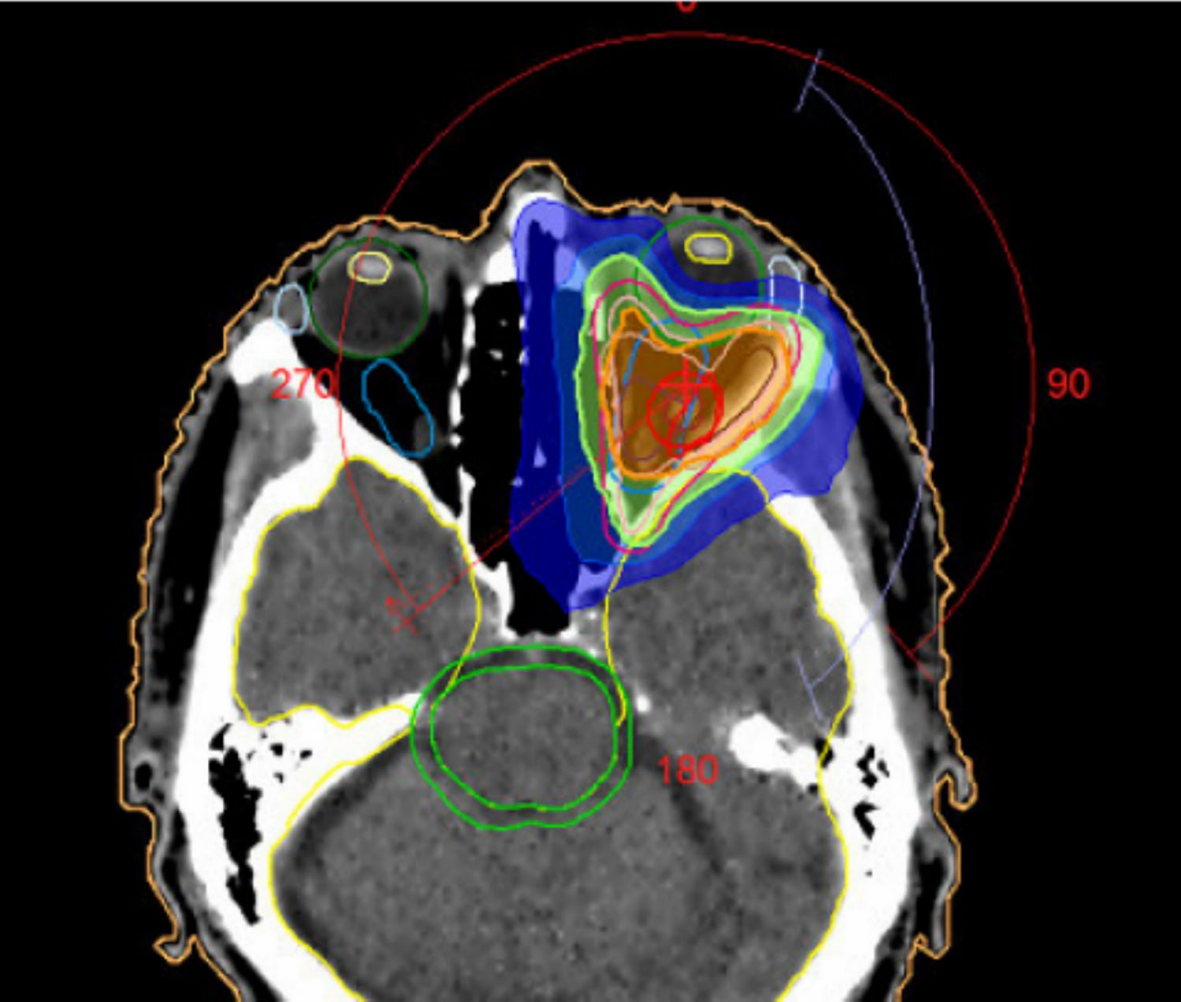
The radiation field targeted to the left orbit, with an additional boost directed to the metastasis

**Fig. 3:**
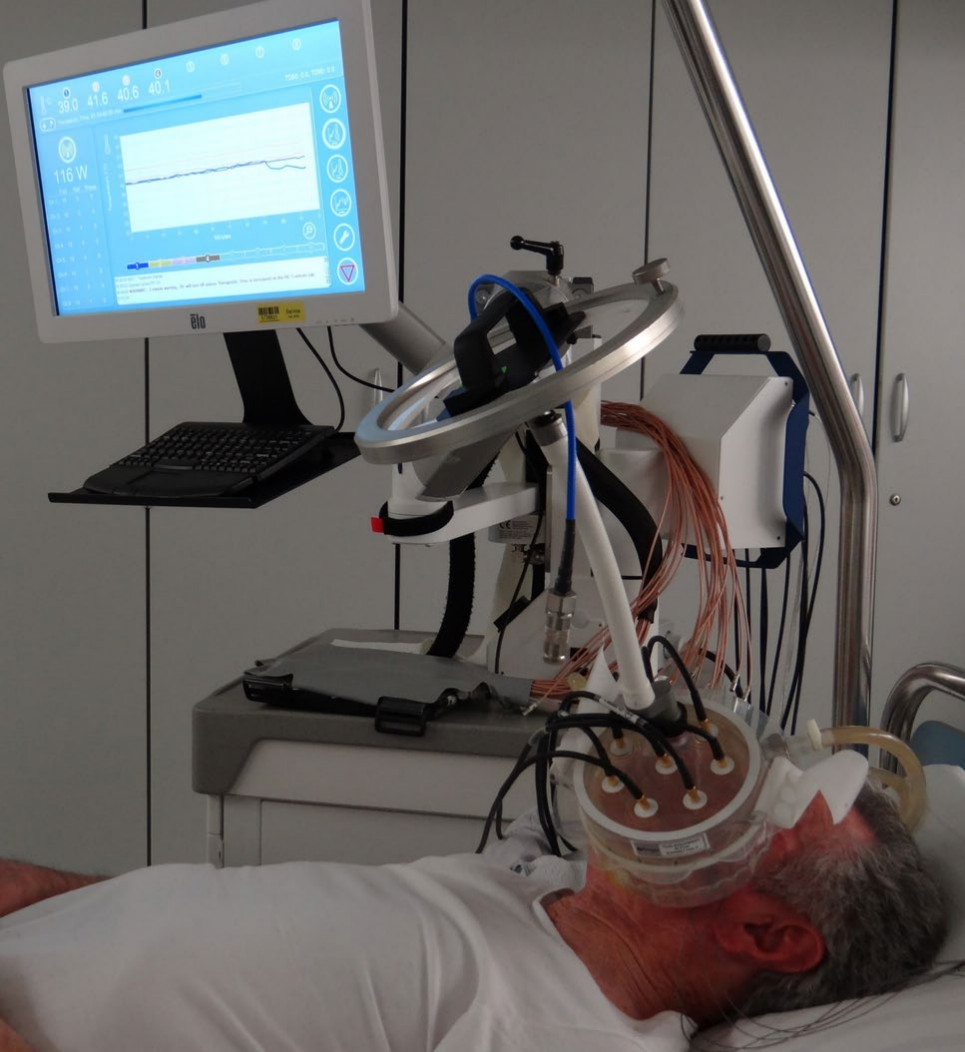
Application of regional hyperthermia with the BSD-500 System (Pyrexar Medical, Salt Lake City, UT, USA)

**Fig. 4:**
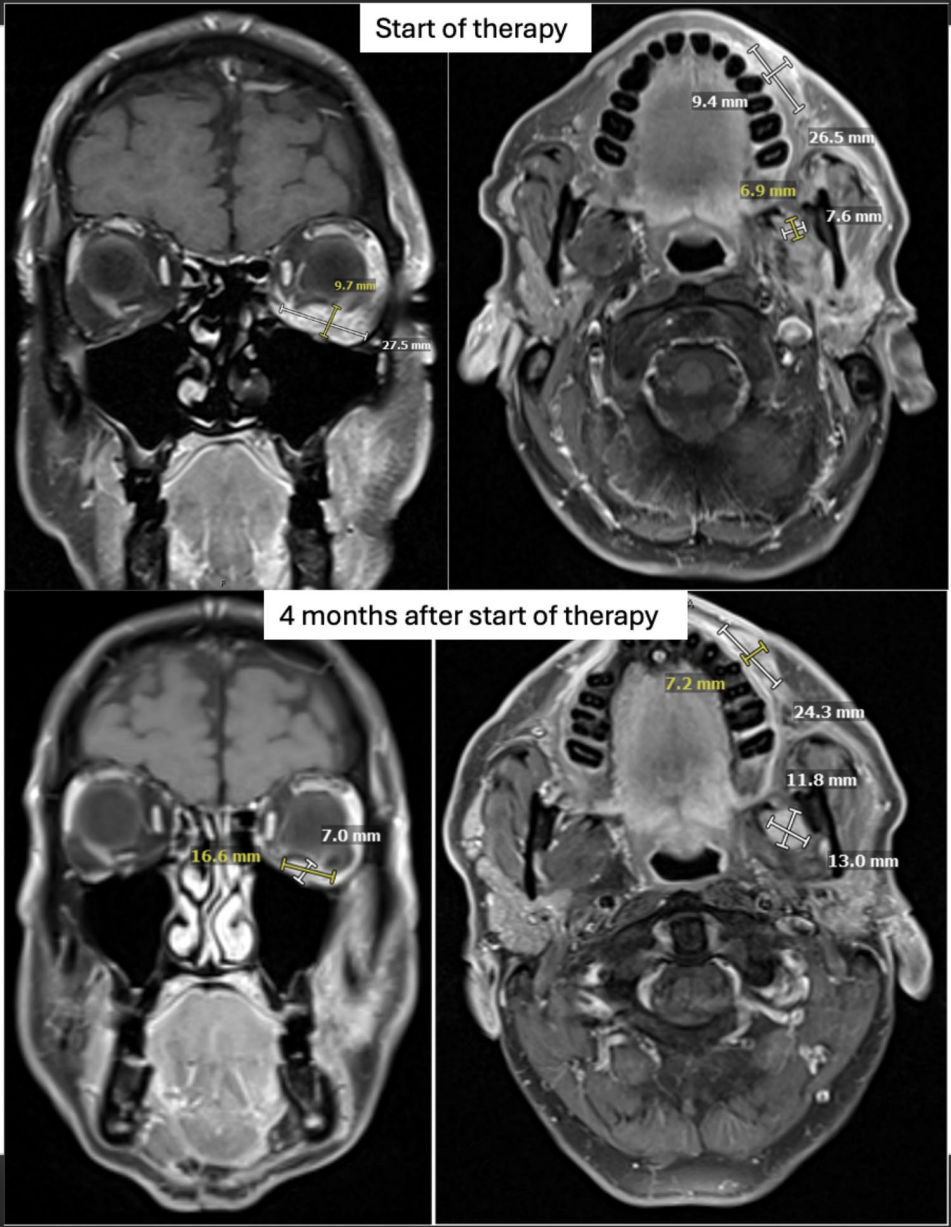
Evolution of metastatic lesions before radiotherapy, nivolumab and regional hyperthermia and 4 months after treatment

**Fig. 5:**
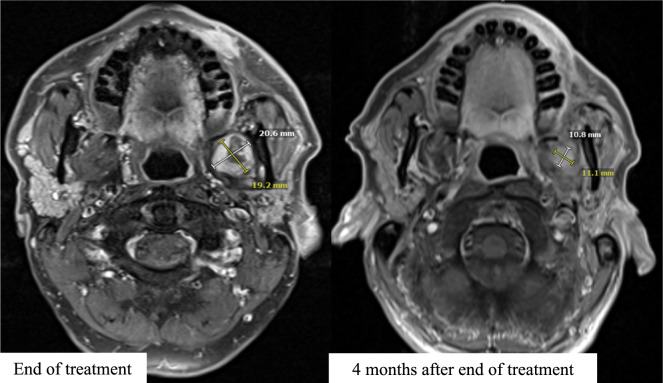
Stabilization of the metastatic lesion in the masticator space over several months after the end of therapy with nivolumab and regional hyperthermia

The complete systemic treatment regimens are illustrated in Fig. 1. A first recurrence occurred approximately 3 years after initial diagnosis, with MRI demonstrating an extensive tumor recurrence which extended laterally and cranially over the parapharyngeal space and the masticator space and infiltrated the parotid gland. Histological work-up was again negative for PD-L1 (TPS 0%, CPS 0, IC-Score 0%). NGS was negative for mutations, fusions or copy number variations. The tumor mutational burden was low (0 Mut/Mb). The patient underwent two sessions of carbon ion irradiation, receiving 2 fractions of 6 Gy (RBE). Treatment was discontinued at the patient’s request, and systemic therapy with paclitaxel (80 mg/m^2^) and cetuximab (250 mg/m^2^ weekly) was initiated. Therapy was first reduced in dose and later deescalated to cetuximab monotherapy due to progressive hematological toxicity. Eight months after beginning of treatment with paclitaxel and cetuximab, a second recurrence occurred with a new intraconal metastasis with infiltration of the orbital fat tissue and rectus muscles in addition to tumor progression in the masticator area. The patient underwent local radiotherapy to the left orbit, receiving a total dose of 20 Gy at 4 Gy per fraction, with a simultaneous integrated boost (SIB) of 5 Gy per fraction to a total of 25 Gy targeting the visible tumor (Fig. 2). Treatment with nivolumab (240 mg every 2 weeks) was started after the end of radiotherapy. Due to the negative PD-L1 expression in this patient, RHT was applied every 2 weeks as an off-label treatment to potentially increase the effect of nivolumab. The heating field was directed on the superficial left masticator space with the BSD-500 hyperthermia system (Pyrexar Medical, Salt Lake City, UT, USA, Fig. 3) over 60 min. The BSD-500 microwave RHT system is equipped with a 915 MHz power solid state generator. The generator features 8 independently adjustable channels for control of phase and amplitude and delivers 400 W of microwave energy. The system is equipped with four different surface applicators for the treatment of superficial tumors of different diameters with a maximum penetration depth of approximately 2.5 cm. At a maximum output of 116 W, skin temperatures of 42.3 °C were achieved in the target area. Quality and safety of hyperthermia was ensured by the European Society for Hyperthermic Oncology (ESHO) guidelines [12]. As the treatment progressed, the patient’s symptoms gradually improved from a clinical perspective. Both eye and jaw movements became increasingly feasible and visible tumor manifestations became less prominent. A first local staging was performed 4 months after beginning of treatment with nivolumab and RHT. MRI showed a partial response of the intraconal metastasis and manifestations in the superficial masticator space. Outside of the RHT field, the satellite metastasis in the deep left masticator space significantly increased in size (Fig. 4). Therapy with nivolumab and RHT was continued due to a continuous clinical benefit. Approximately 9 months after initiation of nivolumab and RHT, another MRI demonstrated progressive metastases in the left maxilla, the left masticator space, and a new metastasis caudal of the left thyroid cartilage. Due to overall significant tumor progression, therapy with nivolumab and RHT was terminated after 17 cycles. The patient underwent radiotherapy for the new lesions using intensity-modulated radiotherapy (IMRT), delivering 4 Gy per fraction to a total dose of 20 Gy. Four months later, despite overall tumor progression, a delayed tumor response was visible in the satellite metastasis in the deep masticator space (Fig. 5). Off-label radioablation of the left orbital metastasis and Fibroblast Activation Protein Inhibitor (FAPI) therapy was initiated on patient request outside of our institution. Due to tumor progression 6 weeks after initiation of FAPI therapy, the patient opted for Best Supportive Care (BSC) and passed away shortly after.

## Discussion

To our knowledge, this is the first case report to demonstrate the feasibility and effectiveness of the combination of nivolumab and regional hyperthermia (RHT) in a patient with recurrent Head and Neck Squamous Cell Carcinoma (HNSCC). Treatment was well tolerated, and tumor control was achieved over 9 months despite negative PD-L1 expression. Tumor progression occurred in areas outside of the RHT field, which suggests a potential synergy between immune checkpoint inhibitor therapy and RHT. Interestingly, there seemed to be a delayed response to nivolumab and RHT in a satellite metastasis in the masticator space, which first increased in size and became partially necrotic before decreasing in size 4 months after the end of therapy. Overall, the response and treatment duration described in our patient was significantly higher compared to current data on immune checkpoint inhibitors in recurrent and metastatic HNSCC: In the landmark paper from Ferris et al. which resulted in the FDA approval for nivolumab in recurrent and metastatic HNSCC, median progression-free survival (PFS) was 2.0 months, and response rate was 13.3% [3]. In the KEYNOTE-012 expansion cohort, response rates were significantly lower in PD-L1-negative patients with recurrent/metastatic HNSCC (4% vs. 22% in PD-L1-positive patients) [6]. This effect was also visible in patients undergoing treatment with the PD-L1 inhibitor durvalumab, with an overall response rate of 2.6% in patients with PD-L1 expression under 25% [4].

RHT is an established treatment modality in combination with chemo- and radiotherapy in several solid tumors [7, 9]. We hypothesize that the addition of RHT renders the tumor immunogenic and, therefore, more susceptible to a treatment approach with immune checkpoint inhibitors [8, 13, 14]. The immunogenic effect of hyperthermia is based on several working mechanisms across multiple cell levels [15]. Hyperthermia results in both active and passive release of tumor antigens and heat shock proteins (HSP), which stimulates downstream immune activity and antigen presentation [16–18]. Additionally, RHT facilitates the migration of antigen-presenting cells (APCs) to lymph nodes and subsequent activation and trafficking of T cells to the tumor area [19, 20]. Moreover, RHT increases blood perfusion in the tumor area, which may facilitate the infiltration of co-stimulatory molecules or immune effector cells [21, 22]. The immunogenic effect of RHT is also derived from direct apoptosis of tumor cells through thermal stress-induced up-regulation of specific cytokines [23]. Preclinical data suggest an increased local and distant tumor control when RHT is added to the treatment with immune checkpoint inhibitors, also described as an abscopal effect [10]. With regard to head and neck cancers, several clinical studies demonstrated a benefit in combining RHT with radiotherapy, which resulted in encouraging response and survival rates [24]. To our knowledge, there are, however, no relevant clinical trials on the combination of chemotherapy or immune checkpoint inhibitors with RHT in HNSCC. More data is needed on the synergistic effect of these treatments in this patient collective.

In addition to the clinical benefit, an important advantage of RHT is the feasibility in a clinical context: RHT has a favorable toxicity profile, with only mild reported toxicities due to the increase in temperature to 42–43 °C [25]. RHT can be safely administered for long time periods as described in our patient, who underwent RHT every 2 weeks for 9 months. Long-term treatment feasibility is becoming more important in the context of immune checkpoint inhibitor therapies, as some patients with favorable responses may undergo treatment for several years [26].

An important limitation of this case report is the concomitant use of radiotherapy (RT). p16-positive HNSCC is thought to be a distinct entity with higher response rates to RT, and several studies suggest de-intensified RT protocols without a decrease in tumor control [27]. In our patient, we postulate that the initial tumor response in the orbital region was mainly attributed to RT. However, the prolonged therapeutic response may be linked to the combination of regional hyperthermia and immunotherapy, especially in this patient with multiple recurrences. This hypothesis is supported by the observation that the deep tissue metastases exhibited an objective response, in contrast with other lesions that showed progression under immunotherapy alone. These findings suggest a potential synergistic effect of RHT and immune checkpoint inhibitors. Prospective studies are needed to address this research question.

## Conclusion

This case reports demonstrates a prolonged response to nivolumab and RHT in a patient with recurrent/metastatic HNSCC and negative PD-L1 expression. We demonstrate the feasibility and clinical potential of the addition of RHT to systemic treatment in this patient collective with dismal outcomes and low response rates to immune checkpoint inhibitors. RHT might be an additional tool to activate an immunogenic milieu responsive to systemic treatment. Prospective clinical trials are needed to address this research question.

## Data Availability

The data presented in this study are available on specific request from the corresponding author. The data are not publicly available for reasons of data protection and data economy.
